# Incisional hernia recurrence through genomic profiling: a pilot study

**DOI:** 10.1007/s10029-012-0923-4

**Published:** 2012-05-31

**Authors:** R. Calaluce, J. W. Davis, S. L. Bachman, M. M. Gubin, J. A. Brown, J. D. Magee, T. S. Loy, B. J. Ramshaw, U. Atasoy

**Affiliations:** 1 Department of Surgery, The University of Missouri Health Sciences Center, University of Missouri, One Hospital Drive, M610C, Columbia, MO 65212 USA; 2 Department of Health Management and Informatics, The University of Missouri Health Sciences Center, University of Missouri, Columbia, MO USA; 3 Department of Statistics, University of Missouri, Columbia, MO USA; 4 Department of Molecular Microbiology and Immunology, University of Missouri, Columbia, MO USA; 5 Department of Pathology, Ross University, Roseau, Dominican Republic; 6 Transformative Care Institute, Daytona Beach, FL USA; 7 Department of Child Health, University of Missouri, Columbia, MO USA

**Keywords:** Ventral hernia, Recurrence, Microarray, *GREM1*, Gene expression, Collagen I/III ratio

## Abstract

**Purpose:**

Although situational risk factors for incisional hernia formation are known, the methods used to determine who would be most susceptible to develop one are unreliable. We hypothesized that patients with recurrent incisional hernias may possess unique gene expression profiles.

**Methods:**

Skin and intact fascia were collected from 15 normal control (NC) patients with no hernia history and 18 patients presenting for recurrent incisional hernia (RH) repair. Microarray analysis was performed using whole genome microarray chips on NC (*n* = 8) and RH (*n* = 9). These samples were further investigated using a pathway-specific PCR array containing fibrosis-related genes.

**Results:**

Microarray data revealed distinct differences in the gene expression profiles between RH and NC patients. One hundred and sixty-seven genes in the skin and 7 genes in the fascia were differentially expressed, including 8 directly involved in collagen synthesis. In particular, *GREMLIN1*, or bone morphogenetic protein antagonist 1, was under expressed in skin (fold = 0.49, *p* < 10^−7^, *q* = 0.0009) and fascia (fold = 0.23, *p* < 10^−4^, *q* = 0.095) of RH patients compared with NC. The PCR array data supported previous reports of decreased collagen I/III ratios in skin of RH versus NC (mean = 1.51 ± 0.73 vs. mean = 2.26 ± 0.99; one-sided *t* test, *p* = 0.058).

**Conclusion:**

To our knowledge, this is the first microarray-based analysis to show distinct gene expression profiles between the skin and fascia of RH and NC patients and the first report of an association between *GREMLIN1* and incisional hernia formation. Our results suggest that gene expression profiles may act as surrogate markers that stratify patients into different groups at risk for hernia development prior to their initial surgery.

**Electronic supplementary material:**

The online version of this article (doi:10.1007/s10029-012-0923-4) contains supplementary material, which is available to authorized users.

## Introduction

Incisional hernia repair comprises a significant proportion of a general surgeon’s practice. The incidence of incisional hernias ranges from 2 to 11 %, with a substantial recurrence rate reported between 10 and 50 % [1]. Based upon this estimate, 100,000 incisional hernia repairs are predicted to be performed each year costing $2.5 billion [1]. While recurrence rates have decreased by using prosthetic mesh in the repair, a significant number of patients develop multiple recurrences with estimates in the literature ranging from 5 to 20 % [2].

Several risk factors for developing incisional hernias have been identified including wound infection, abdominal distention, pulmonary complications, male gender, age, and obesity [1]. Although risk factors for recurrent incisional hernias have also been evaluated, the literature is controversial with regard to many of these, such as body mass index, ascites, large hernias exceeding 10 cm in width or length, continued smoking, occupational lifting, and wound-healing disorders (e.g., hematoma, seroma, infection) [1].

Current data suggest that incisional hernias are commonly caused by failure of early surgical wound healing [3]. Since collagen I provides tensile strength to connective tissue, and immature collagen III found in early wounds is weaker, investigations into the collagen I-to-III ratio have demonstrated a decreased ratio in patients with direct and indirect hernias as compared with controls [4, 5]. This decrease in the collagen I/III ratio was attributed to the relative increase in collagen III synthesis and was also seen in incisional hernias [4, 6]. Moreover, a decreased collagen I/III ratio in incisional hernias supports the possibility of a high-risk group more susceptible to hernia formation [7]. White and colleagues performed a preliminary immunohistochemical trial examining the skin and fascia of 16 incisional hernia patients for collagen I and III and compared the ratio to normal foregut collected from bariatric patients [8]. They found a significant decrease in the ratio in the skin of the hernia patients but found no difference in the fascia [8].

While patients with collagen and connective tissue diseases, such as Ehlers–Danlos syndrome, osteogenesis imperfecta, and Marfan’s syndrome, are known to form hernias, there are no data on potential genetic predispositions to hernia formation in otherwise normal patients [9–11]. We hypothesized that recurrent incisional hernia formation may be due to subtle differences in gene expression (mRNA) profiles that ultimately alter wound healing. We designed a pilot study comparing the skin and fascia from recurrent hernia (RH) patients to those who underwent laparoscopic cholecystectomy (normal control, NC) in order to identify distinct genomic profiles in the two patient populations.

## Methods

### Patient samples and tissue acquisition

After obtaining IRB approval and receiving appropriate informed consent, 33 patients participated in this study. Patients were eligible if they were 18 years of age or older and underwent laparoscopic repair of a recurrent ventral or incisional hernia. Patients were excluded if they were under 18; had a history of steroid use, severe COPD, pulmonary, or connective tissue disorders; or were prisoners. Eighteen patients with at least one recurrent incisional hernia presented for laparoscopic incisional hernia repair. The designated controls were 15 healthy patients who had no hernia history and underwent laparoscopic cholecystectomy. Approximately 1 cm^2^ of skin and fascia was removed from the trocar placement site, remote from the hernia or old incisions. The tissue samples were divided and placed in either 10 % buffered formalin or RNALater™ RNA Stabilization Reagent (Qiagen, Valencia, CA). Tissue was stored in RNALater™ for up to 48 h at room temperature. Approximately 100–150 mg of tissue was used for RNA isolation.

### RNA isolation and RNA amplification

Total RNA was isolated from the skin and fascia specimens by following the manufacturer’s protocol from the RNeasy^®^ Lipid Tissue Mini Kit (Qiagen) using a rotor homogenizer and on-column DNase treatment. Total RNA was amplified using the WT-Ovation™ Pico RNA Amplification System protocol (NuGen, San Carlos, CA) as previously described [12, 13].

### cDNA labeling, RNA quantity and quality, and microarray

Of the 33 enrolled patients, 8 NC and 9 RH patients were selected for microarray analysis based on the quantity, quality, and integrity of the RNA. For each skin and fascia sample, 1.5 μg biotin labeled, amplified cDNA was hybridized to a Sentrix^®^ Human-6 v.2 Whole Genome Expession BeadChips (Sentrix Human WG-6; Illumina, San Diego, CA) as previously described [13].

### Validation by quantitative RT-PCR (qPCR) and PCR array

cDNA was generated from 10 ng of the same total RNA samples as used for the microarray experiment (15 patients analyzed by microarray with sufficient amounts of remaining high-quality RNA) and SuperScript™ III Platinum^®^ Two-Step qPCR Kit with SYBR^®^ Green (Invitrogen Carlsbad, CA). For *COL1A* and *GREM1*, qPCR was performed on the StepOne™ Real-Time PCR System (Applied Biosystems, Foster City, CA) using *GAPDH* as a reference gene as previously described [13]. A PCR array, focusing on the expression of 84 key genes related to dysregulated tissue remodeling during wound healing, was also performed on these 15 patients by Global Biologics (Columbia, MO). Briefly, RNA quantity and purity were assessed using NanoDrop ND-2000 (Nanodrop Technologies, Wilmington, DE, USA). RNA integrity was evaluated using the RNA integrity algorithm generated by the Bioanalyzer 2100 with the Eukaryotic RNA Pico Series II reagents (Agilent Technologies, Santa Clara, CA, USA). RIN values ranged from 5 to 8. RNA was reverse transcribed with the RT^2^ First Strand cDNA kit (SABiosciences, Frederick, MD), and qPCR was performed using the Human Fibrosis RT^2^ Profiler™ PCR Array System (SABiosciences, Frederick, MD) and the Roche LightCycler480 instrument. As part of the qPCR quality assessment process, each sample was evaluated for the presence of genomic cDNA contamination, followed by three positive PCR and three reverse transcriptase controls. The chosen housekeeping or reference gene, *RPL13A*, was selected from a panel of five housekeeping genes on the array based on the most uniform expression range across all samples. *GREM1* and *COL1A* qPCR data were statistically compared using a two-sample *t* test on the ∆*C*_t_ values. The PCR array data were compared between groups using a moderated *t* test on the ∆*C*_t_ values as long as the gene was considered to be reliably expressed (*C*_t_ < 35 in 75 % of samples) [14].

### Immunohistochemistry

Specimens were fixed in 10 % buffered formalin, routinely processed, embedded in paraffin, and cut at 4 μm. Immunohistochemistry was performed using the automated horseradish peroxidase Autostainer/Envision Plus method (Dakocytomation, Carpenteria, CA) as previously described [15, 16].

### Statistical analysis of microarray data

Analysis of microarray gene expression data was primarily performed using R open-source software (R Foundation, Vienna, Austria). Any genes considered “not detectable” (Illumina software detection <1 %) across >50 % of patient samples were excluded from further statistical analyses in order to reduce false positives. Nonspecific filtering was also carried out to remove genes with little variability as previously described [17]. Differential gene expression analysis was performed using a moderated *t* statistics applied to the log_2_-transformed normalized intensity for each gene using an empirical Bayes approach [14]. Adjustment for multiple testing was made using the false discovery rate method of Benjamini and Hochberg with a significance cutoff of *q* < 30 % [18], since the list of discovered genes was relatively small. We declared a gene differentially expressed if it was statistically significant after adjusting for multiple testing and had a fold change ≥ 1.5 (either over- or under expressed).

Gene ontology (GO) analyses were conducted on the resulting list of significantly different genes to test their association with independently established GO terms to shed insight on the common functions of the differentially identified genes. We carried out GO analyses for overrepresentation of biologic process, molecular function, and cellular component ontologies, which generated an odds ratio (OR) and *p* value for each GO category, using methods previously described [13]. A small *p* value (<0.05) and large OR indicated that the number of selected genes associated with a given term (e.g., wound healing) was larger than expected due to chance. GO categories containing less than 10 genes represented on the array were not considered to be statistically reliable indicators and were not reported even if significant.

## Results

### Demographics

Demographics for the 33 enrolled patients and the subset of 17 patients whose samples were analyzed by microarray are shown in Table 1. The majority (26/33) of enrolled patients were female, and all but one sample analyzed by microarray were from females. The RH and NC groups analyzed by microarray were comparable (*p* > 0.05) on all demographics except diabetes (*p* = 0.03) and previous surgery (*p* = 0.01), neither of which is unexpected in these populations.

**Table 1:** Demographics of enrolled patients and the subset analyzed by microarray

Characteristics	Patients enrolled			Patients analyzed by microarray		
	RH (*n* = 18)	NC (*n* = 15)	*p*	RH (*n* = 9)	NC (*n* = 8)	*p*
Sex (M/F)	4/14	3/12	0.99	0/9	1/7	0.47
Age	553.2	44.9	0.14	50.9	39.1	0.23
BMI	36.6	30.5	0.03	39.2	31.4	0.10
Smoker	8	2	0.07	4	2	0.62
Diabetes	7	0	0.01	5	0	0.03
Previous surgery	18	6	0.01	9	3	0.01

### Identification of differential gene expression in the skin and fascia of RH patients via microarray

Illumina microarray data revealed that 142 complete genes and 25 expressed sequence tags (ESTs) for a total of 167 genes were differentially expressed in the skin, and 6 complete genes and 1 EST were differentially expressed in fascia for a total of 7 genes. While the full results are included in Online Resources 1 and 2, a representative list of genes is reported in Tables 2 and 3. These were selected based on our interest in hernia formation and wound healing, as well as regulation of transcription and immunology.

**Table 2:** Selected genes from skin of RH patients significantly over- or under expressed in comparison with skin from NC, in ascending order of fold change (NC/RH)

Gene symbol	Fold change	Gene name
*GREM1*	0.49	Gremlin 1, cysteine knot superfamily, homolog (*Xenopus laevis*)
*TP63*	0.5	Tumor protein p63
*KRT15*	0.53	Keratin 15
*TFAP2C*	0.59	Transcription factor AP-2 gamma (activating enhancer binding protein 2 gamma)
*KLF5*	0.63	Kruppel-like factor 5 (intestinal)
*ELL2*	0.66	Elongation factor, RNA polymerase II, 2
*NAP1L1*	0.66	Nucleosome assembly protein 1-like 1
*COL5A2*	1.51	Collagen, type V, alpha 2
*PDXK*	1.51	Pyridoxal (pyridoxine, vitamin B6) kinase
*GHR*	1.54	Growth hormone receptor
*NUCB1*	1.55	Nucleobindin 1
*CD81*	1.56	CD81 molecule
*RBPMS2*	1.59	RNA binding protein with multiple splicing 2
*TIMP1*	1.59	TIMP metallopeptidase inhibitor 1
*ANXA5*	1.59	Annexin A5
*CAV1*	1.60	Caveolin 1, caveolae protein, 22 kDa
*THY1*	1.62	Thy-1 cell surface antigen
*PMP22*	1.62	Peripheral myelin protein 22
*COL5A1*	1.63	Collagen, type V, alpha 1
*FBLN1*	1.63	Fibulin 1
*FBN1*	1.63	Fibrillin 1
*CLDN5*	1.66	Claudin 5 (transmembrane protein deleted in velocardiofacial syndrome)
*MSX1*	1.66	Msh homeobox 1
*COL1A2*	1.69	Collagen, type I, alpha 2
*PDGFRB*	1.7	Platelet-derived growth factor receptor, beta polypeptide
*FAP*	1.74	Fibroblast activation protein, alpha
*DCN*	1.74	Decorin
*MCAM*	1.79	Melanoma cell adhesion molecule
*COL6A3*	1.8	Collagen, type VI, alpha 3
*CILP*	1.99	Cartilage intermediate layer protein, nucleotide pyrophosphohydrolase
*LUM*	2.03	Lumican
*COL1A1*	2.05	Collagen, type I, alpha 1
*FZD4*	2.11	Frizzled homolog 4 (Drosophila)
*CTHRC1*	2.17	Collagen triple helix repeat containing 1
*HSPB6*	2.17	Heat shock protein, alpha-crystallin-related, B6
*RBP4*	2.17	Retinol binding protein 4, plasma
*COL3A1*	2.3	Collagen, type III, alpha 1
*COL4A1*	2.43	Collagen, type IV, alpha 1
*ANGPTL2*	2.7	Angiopoietin-like 2
*CD36*	3.07	CD36 molecule (thrombospondin receptor)
*FSTL1*	3.15	Follistatin-like 1
*PCOLCE2*	3.64	Procollagen C-endopeptidase enhancer 2
*LEP*	5.03	Leptin

**Table 3:** Selected genes from fascia of RH patients over- or under expressed in comparison with fascia from NC patients in ascending order of fold change (NC/RH)

Gene symbol	Fold change	Gene name
*GREM1*	0.23	Gremlin 1
*PRLR*	0.39	Prolactin receptor
*LEFTY*	0.43	Left–right determination factor
*SCRG1*	0.44	Scrapie responsive protein 1
*RNF144A*	0.49	Ring finger protein 1
*PDZRN4*	0.54	PDZ domain containing ring finger 4

Eight discovered genes were directly involved in collagen synthesis (*PCOLCE2*, *CTHRC1*, *COL1A1*, *COL3A1*, *COL4A1*, *COL5A1*, *COL5A2*, and *COL6A3*). Moreover, as supported by the literature, several have been associated with hernia formation, Ehlers–Danlos syndrome, and Marfan’s syndrome (e.g., *COL1A1*, *COL3A1*, *COL5A1*, *FBN1*, and *TIMP1*).

A novel and unexpected gene found to be statistically significant in both the skin and fascia was *GREMLIN1* (*GREM1*, also known as cysteine knot superfamily 1, BMP Antagonist 1, *CKTSF1B1*; induced in high glucose 2, *IHG*-2; and down regulated by *v*-*mos*, *DRM*) [19]. In fascia, *GREM1* had a fold change of 0.23 (*q* = 0.095, *p* < 10^−4^), while in skin, it was found to have a fold change of 0.49 (*q* = 0.0009, *p* < 10^−7^). *GREM1* was under expressed in both the skin and fascia of RH patients in comparison with NC.

### Gene ontology analysis of differentially expressed genes

Gene ontology analyses were performed to determine whether there were common functions or descriptive terms that were statistically abundant in the list of differentially expressed genes, as quantified by odds ratios. Although the fascia gene list was too sparse for analysis, in skin we found more than 53 biologic process (BP) enriched terms, 18 enriched molecular function (MF) terms, and 10 cellular component (CC) terms (Online Resources 3, 4, and 5).

Table 4 represents a sample of important biologic processes that we found to be differentially enriched in skin. For example, in the skin of RH patients, many differentially expressed genes were found to be more abundant than expected in biologic processes such as: response to wounding; regulation of immune response; activation of plasma proteins during acute inflammatory response; lipid metabolic process; multicellular organismal development; and cell adhesion. Moreover, these analyses illustrate that many genes such as the collagen genes have diverse functions and appear in several BP categories. For instance, *COL3A1* and *FBN1* were associated with response to wound healing, blood coagulation, regulation of body fluids, as well as organ development. *COL3A1* was also associated with regulation of immune response, regulation of multicellular organismal process, negative regulation of response to stimulus, cell–matrix adhesion, and negative regulation of immune system process.

**Table 4:** Selected results from GO analysis of biologic processes in list of differentially expressed genes from skin samples

GO ID	OR	*p*	Term	Differentially expressed genes in term
0002541	7.01	0.039	Activation of plasma proteins involved in acute inflammatory response	*CFD*, *CFH*
0007160	6.62	0.001	Cell–matrix adhesion	*COL3A1*, *ECM2*, *NID1*, *EPDR1*, *THY1*
0050776	4.89	0.012	Regulation of immune response	*COL3A1*, *CFD*, *CFH*, *THY1*
0009611	3.18	0.001	Response to wounding	*COL3A1*, *CFD*, *FABP4*, *FBN1*, *CFH*, *ANXA5*, *PROK2*, *VWF*, *CAV1*, *AOC3*, *CD36*
0007155	2.36	0.007	Cell adhesion	*FERMT2*, *COL5A1*, *COL6A3*, *VCAN*, *DPT*, *ISLR*, *LAMA4*, *MCAM*, *MFAP4*, *S100A4*, *CLDN5*, *AOC3*, *CD36*

These terms are more abundant than expected and are sorted by odds ratio (OR)

### Validation of gene expression by qPCR and PCR array

**Fig. 1:**
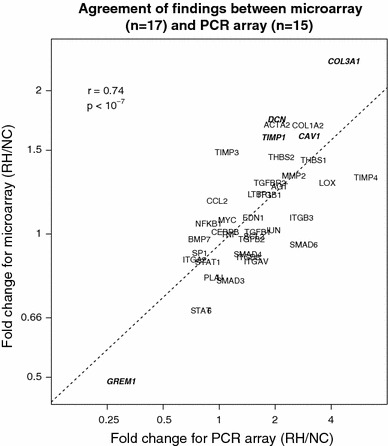
Agreement of microarray and PCR array results. The genes which were detected on both the microarray and the PCR array are plotted against their fold change (RH/NC) for each platform. *Bold italicized gene symbols* indicate they were significantly different based on microarray data

**Fig. 2:**
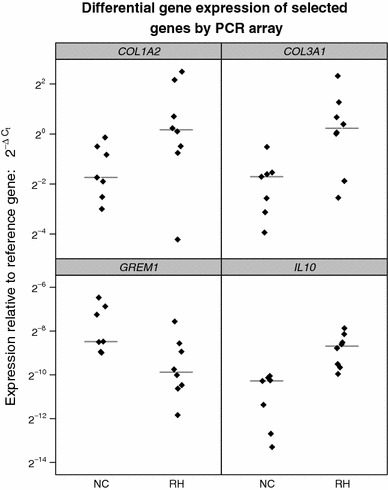
Patient-level gene expression data for 4 selected genes from PCR array with group median indicated by a *horizontal line*

Based upon the Illumina microarray results, *COL1A1* and *GREM1* were selected for validation by qPCR. *COL1A1* was overexpressed (2.33 fold) in the skin of RH patients as compared to NC, but was under expressed (0.34 fold) in the fascia. *GREM1* was under expressed in both the skin (2.6 fold) and fascia (11.2 fold) of RH patients in comparison with NC (Online Resource 6). In order to explore the relationship between other relevant wound-healing genes, such as *COL1A1* and *COL3A1*, a PCR array was used to measure gene expression on a subset of 15 remaining patient samples. Eighty genes on the PCR array were reliably expressed and were analyzed for differences. The PCR array results confirmed the microarray data as illustrated by the strong agreement of fold change (Pearson *r* = 0.74, *p* < 10^−7^) among the 39 genes common to both arrays which were detectable (Fig. 1). The 22 genes with large fold changes found by PCR array are reported in Table 5, with results for all genes on the PCR array presented in Online Resource 7. The distributions of patient expression levels from the PCR array for four selected differentially expressed genes overlap less than 50 % on average (Fig. 2).

**Table 5:** Genes sorted by fold change (RH/NC) in skin by PCR array with fold changes >2 or <0.5 between RH (*n* = 8) and NC (*n* = 7), where * denotes *p* < 0.05

Gene symbol	Fold change	*p*
GREM1*	0.29	0.007
*AGT*	2.08	0.184
*THBS2*	2.11	0.081
*TIMP2*	2.29	0.059
*HGF*	2.32	0.084
*ENG*	2.37	0.060
*MMP2*	2.47	0.054
MMP9*	2.57	0.037
CTGF*	2.65	0.025
ITGB3*	2.72	0.036
*MMP3*	2.78	0.063
SMAD6*	2.78	0.033
COL1A2*	2.92	0.035
CAV1*	2.98	0.020
CCL3*	3.01	0.020
THBS1*	3.15	0.012
SERPINE1*	3.23	0.013
ITGA1*	3.34	0.010
LOX*	3.76	0.006
COL3A1*	4.54	0.002
IL10*	4.83	0.001
TIMP4*	6.02	0.001

### COL1/COL3 ratio by microarray, PCR array, and immunohistochemistry

By microarray, *COL1A1*/*COL3A1* ratio in skin of RH patients was slightly lower than NC patients, but was not significant (1.33 vs. 1.46, *p* = 0.65). Similar but significant results were found for *COL1A2*/*COL3A1* (0.59 vs. 0.79, *p* = 0.02). Neither of these ratios were statistically different in the fascia. Immunohistochemistry on 5 patients demonstrated slightly greater staining intensity of *COL3A1* than *COL1A1* in the skin and fascia from RH patients in comparison with NC. Analysis by PCR array revealed that gene expression of *COL3A1* was greater than *COL1A2* (the second alpha chain of the collagen 1 molecule) in skin in both groups. According to the manufacturer, *COL1A2* was selected because it was referenced more often in relation to fibrosis in public data bases than *COL1A1*. Moreover, the ratio of *COL1A2*/*COL3A1* was decreased in the RH group as compared to NC (1.51 vs. 2.26, *p* = 0.058, one-sided *t* test). These results agree with reports in the literature [4–12].

**Fig. 3:**
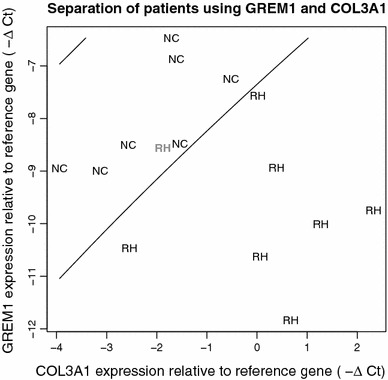
Ability of the combination of *GREM1* and *COL3A1* gene expression to separate RH and NC patients. PCR array data were used to explore the utility of gene expression of *GREM1* and *COL3A1* as markers to distinguish RH and NC patients. The best separation boundary (*solid line*) was determined using quadratic discriminant analysis. Using all of the data, only 1 patient (RH, *gray*) was misclassified (93 % accuracy). Using leave-one-out cross-validation, in which each patient’s data is held out (in turn) during the calculation of the best boundary and subsequently evaluated for accuracy, 13/15 (86 %) patients were correctly classified

The gene expression ratio of *COL1A2*/*COL3A1*, in conjunction with *GREM1*, was explored as a means of stratifying patients into NC or RH. We also considered *COL1A2* and *COL3A1* on their own (i.e., not in ratio form) in combination with *GREM1*. All pairwise combinations of these 4 markers were considered as means of classifying patients into their correct group (RH or NC) using quadratic discriminant analysis (QDA). QDA may be thought of as a method that yields the best curve (“separation boundary”) that can be drawn in order to maximize the separation between the group means. We found that by using leave-one-out cross-validation, the combination of {*GREM1*, *COL3A1*} (Fig. 3) achieved the highest accuracy (86 %), followed by either {*COL3A1*, *COL1A2*} or {*COL1A2*, *COL1A2*/*COL3A1*} at 73 % accuracy, and {*GREM1*, *COL1A2*} or {*GREM1*, *COL1A2*/*COL3A1*} at 66 % accuracy.

## Discussion

The molecular biology of hernia repair is largely unknown. Equally unclear is why incisional hernia repairs, either laparoscopic or open, frequently recur. We designed a pilot study, using microarrays, to identify potentially specific gene profiles in patients with recurrent incisional hernias (RH). We analyzed the skin and fascia from these patients and compared them to skin and fascia taken from patients who had no history of hernias (NC).

Our study was unique both in using a genomic-based approach (microarray and PCR array) and in taking skin and fascia samples away from the site of the incisional hernia. The acquisition of skin and fascia at the start of the procedure, prior to trocar placement, allowed us to avoid the confounder of biologic and pathologic processes occurring in the hernia (e.g., inflammation, wound healing) that could skew our results. Wound infection, for instance, has been widely reported as the most significant independent prognostic factor for incisional hernia [1, 20–22]. Although technical factors such as type of repair or use of mesh have been attributed to cause recurrence, they do not explain all hernia recurrences [1]. We theorized that variations in gene expression may play a role in wound healing and recurrence.

Our experiments have shown distinct gene expression profiles between the skin and fascia of RH and NC patients. When comparing active gene expression profiles, we found more statistically significant genes in the skin than the fascia. We found greater variability in gene expression in fascia than skin in our samples, which is apparent graphically (Online Resources 8 and 9). Since an increase in variance reduces the power to detect differences, this is the most obvious explanation for the shorter fascia gene list. The functions of the genes in the skin were diverse and included wound healing, transcription regulation, and immunology.

The sparse number of genes in the fascia precluded GO analysis. In the skin, GO analysis further expanded these to 53 BP functions, including regulation of the immune and inflammatory responses, organ development, and cell adhesion. GO analysis also revealed 10 CC and 18 MF categories, with most genes associated with the extracellular region and plasma membrane, and enzyme inhibitor activity and receptor binding, respectively. The relationship of these genes to known biologic functions can assist in our understanding of the basic science of hernia formation.

One of our most intriguing findings was altered *GREM1* expression in the skin and fascia of RH patients. Originally isolated from the neural crest of the Xenopus as a bone morphogenetic protein (BMP) antagonist, *GREM1* is an important regulator of limb development and may play a role in regulating organogenesis, body patterning, as well as tissue differentiation [19, 23, 24]. High levels have been found in nondividing and terminally differentiated cells such as neurons, alveolar epithelial cells, and goblet cells [19, 24]. An earlier name of *GREM1* was *IHG*-*2* because its expression in glomerular mesangial cells was induced by high glucose, mechanical strain, and TGF-β [25]. *GREM1* has been suggested to be a modulator of mesangial cell proliferation and epithelial–mesenchymal transdifferentiation in diabetes and has been shown to have increased expression in various diabetic nephropathy models as well as being involved in the pathophysiology of progressive renal fibrogenetic diseases [26, 27]. Moreover, gene and protein expression have been reported in fibroblast cultures harvested from patients diagnosed with systemic sclerosis [28].

Although *GREM1* has not been associated with hernia formation or wound healing, it has been found in the stromal cells of basal cell carcinomas [29]. This group also reported a concomitant expression of *FOLLISTATIN* (*FST*) in the stromal cells of basal cell carcinomas [29]. Interestingly, our data showed that *FST*-*like 1* expression accompanied *GREM1* expression in the skin of recurrent incisional hernia patients. The findings in the literature support a role for *GREM1* in fibrosis of the skin and kidney and are suggestive of a role in hernia formation. The potential role of *GREM1* becomes further substantiated when viewed from a perspective that defects in normal wound healing and mechanical strain are frequently cited as causes of hernia formation and recurrence. Although our microarray data were validated by qPCR and PCR array, we are in the process of further testing the role of *GREM1* in an expanded population of patients.

More conventional genes of interest from our study were the 8 genes directly involved with collagen synthesis and those associated with hernia formation, Ehlers–Danlos syndrome, and Marfan’s syndrome such as *FBN1*. Our data on *COL1A1* and *COL3A1* were validated by qPCR and PCR array. The ratio of collagen I to collagen III decreased in the RH patients in comparison with NC as would be expected according to the literature [4–8]. These data are strengthened by the fact that a decrease was seen regardless of which collagen 1 alpha chain was analyzed. The clinical manifestations of Marfan’s suggest that alterations in connective tissue stability may play an important role. Mutations in *FBN1* are known to cause Marfan’s syndrome and have been associated with tissue stability [30]. Recently, an immunohistochemcial study was performed on scar and nonscar regions of human skin and fascia [30]. The authors studied 22 patients who underwent repeated laparotomy: 12 had developed incisional hernia and 10 did not and were used as control. They found that *FBN1* may be an important contributing factor to tissue stability and incisional hernia formation [30].

Although the size of our study may be viewed as a potential limitation, it is important to emphasize that this was a limited pilot study to assess the feasibility of whether recurrent incisional hernia formation is due to differing gene expression profiles that alter wound healing. The statistical power generated from a larger study that could incorporate adjustments for demographic variables should further substantiate our results. These data would be used to enhance our knowledge of the molecular biology of hernia formation and wound healing. In addition, the gene expression profiles would have both narrow and broad ramifications. For instance, they could predict which ventral hernia patients might be more likely to recur, and potentially offer targeted, patient-specific therapies for the prevention of recurrent incisional hernia such as type of mesh and repair method. A limited investigation of this possibility showed that the combination of *GREM1* and *COL3A1* have potential in this regard (Fig. 3). It is not surprising, since *COL1A2*, *COL3A1*, *GREM1*, and *IL10* all show promise as biomarkers (either individually or more powerfully in the form of a panel) due to the minimal overlap in the RH and NC groups for each of these genes (Fig. 2). From a broader vantage point, however, profiles could be generated from a preoperative assay that would also stratify patients into low- and high-risk populations of prospective hernia formers or poor wound healers. Ultimately, this would lead to the development of rapid and standardized wound-healing methods providing minimal or no postoperative complications.

## Conclusion

In summary, using microarray analysis, we have performed for the first time a genome-wide pilot study of patients who have recurrent incisional hernias. We have identified distinct gene expression profiles in these patients and have furthered our understanding of recurrent incisional hernia formation. Moreover, we have found an association between a novel gene to the hernia literature, *GREM1*, and incisional hernia formation. To our knowledge, this is the first report to demonstrate such an association. Based upon our results, gene expression profiles may act as surrogate markers that stratify patients into different groups at risk for hernia development prior to their initial surgery. Further investigation using a larger patient population is planned to substantiate these results and potentially provide novel insights into hernia formation, wound healing, and ultimately targeted, patient-specific therapy.

## Electronic supplementary material

Below is the link to the electronic supplementary material.

Supplementary material 1 (PDF 1008 kb)

## Abbreviations

RT-PCR: Reverse-transcriptase polymerase chain reaction
*C*_t_: Cycle threshold
cDNA: Complementary DNA
UNG: Uracil-*N*-glycosylase
IRB: Institutional review board
OR: Odds ratio
GO: Gene ontology
RIN: RNA integrity number
