# Supplementary material for: Incisional hernia recurrence through genomic profiling: a pilot study
Source: Hernia. 2012 May 31;17(2):193–202. doi: 10.1007/s10029-012-0923-4 (PMC3606513; doi:10.1007/s10029-012-0923-4)

Online Resource 1
List of differentially expressed genes in skin (q<30%) from microarray, fold=RH/NC.

RefSeq	nuID	Symbol	AveExpr	p	q	Fold
NM_004004	cjif7cfQC.v58VfSXU	GJB2	10.08	0	0.14	0.38
NM_002638	NeHlSg0ILnuCnfmo6U	PI3	8.45	0.01	0.29	0.46
NM_013372	6qgEA34VJ4E7fXi0uU	GREM1	8.38	0	0.09	0.49
NM_001126128	3fSd576nru6EgoOgJQ	PROK2	9.47	0	0.09	0.49
NM_012153	xKpN5Q93R7r193h.l4	EHF	11.38	0	0.2	0.49
NM_001114978	3JikV8enxddOklx3X4	TP63	10.1	0	0.24	0.5
	l7R7dcEsfJLt45NEql		8.18	0	0.08	0.51
NM_007231	WkSP0Ei5_kz7KudDro	SLC6A14	9.47	0	0.21	0.51
NM_002275	N_SiAbV_UdVHdNKULo	KRT15	10.63	0	0.09	0.53
NM_006025	3_1VBVHiBwOexRIlq4	P11	10.1	0	0.11	0.54
NM_005518	cpX09d1OeQ0e16nzrk	HMGCS2	8.93	0.01	0.26	0.54
	0XR.jqKIeSs6iSylfg		8.08	0	0.08	0.55
NM_144670	iqp3UiKla6yKuByIlw	A2ML1	9.25	0	0.17	0.55
	E.euXOJyc0XF4Cuflo		11.11	0	0.23	0.56
NM_004925	in3Ep.6gsq6O6sqeoo	AQP3	12.49	0.01	0.26	0.56
NM_005792	ilUo8I6OXwmOlSq5_s	MPHOSPH6	9.62	0.01	0.27	0.56
	ifuT0uCbzzlEBwXF0U		8.77	0	0.14	0.57
	iXogXCHUAG6x6KLoR4		9.75	0	0.14	0.57
	WkCMln1QI16IFwh1AI		9.53	0	0.17	0.57
NM_003045	EdV._eEEe7E_FH1xTE	SLC7A1	10.63	0	0.2	0.58
NM_005127	oEqyEpSqACkdKkRJPk	CLEC2B	9.61	0	0.21	0.58
NM_020639	K.qs6qi794Xn_SG5dY	RIPK4	9.99	0	0.19	0.59
NM_003222	ZYJD__eftFVC1W6K94	TFAP2C	10.52	0.01	0.29	0.59
NM_006919	l4Wolmt25zei1xEKX8	SERPINB3	8.08	0.01	0.27	0.61
	TdINSqx0pHob6N0k0k		7.96	0	0.08	0.62
	NLi56u6yjuR59cd17o		8.75	0	0.11	0.62
NM_001040101	B_mSa7k0im7niCrrvA	D4S234E	11.58	0	0.14	0.62
NM_080474	6lHqnvt2gK0cY36ui8	SERPINB12	9.35	0	0.23	0.62
	fqfEtK7Q_0sG5X4fUo		8.11	0	0.13	0.63
	NVfUXd.l7KOx7Oy05E		9.18	0	0.16	0.63
NM_173515	05P_F70u3t9SRruxH0	CNKSR3	9.18	0	0.21	0.64
NM_019852	cefr67QKA1UKfQUq3o	METTL3	10.06	0	0.21	0.64
	N07Ve69f_q_G.6qe.U		9.94	0	0.24	0.64
NM_004869	9q37X0X.C9KgS.teFE	VPS4B	10.86	0.01	0.25	0.64
NM_020168	fKYFF1q.U07QrRKTU4	PAK6	8.67	0.01	0.27	0.64
	9oJYvTQ.7gg_fejh4U		9.18	0.01	0.27	0.64
	NenOs7LA6pXszqPlBU		9.23	0	0.2	0.65
NM_000222	ulvN6CwU.kei9zndkU	KIT	8.69	0	0.11	0.66
	r4h0auSuTKgp69e1Kg		9.46	0	0.2	0.66

	WZNV5dTXLAh1_c55J4		9.76	0	0.2	0.66
NM_018686	Hjgi5_ClupcLpnXnjk	CMAS	10.52	0	0.21	0.66
NM_001114106	0odOn.BxDak9Su6ktU	SLC44A3	8.36	0	0.23	0.66
NM_018290	3rfEI9QDB1L6kVOJyg	PGM2	9.2	0.01	0.25	0.66
NM_004537	lrfoO4ET7_y5zU9d14	NAP1L1	9.61	0.01	0.27	0.66
NM_033505	fSrop9yt5OOOqVv3pQ	SELI	9.19	0	0.2	0.67
NM_001864	uFlaBZ.SBYuZiASCd8	COX7A1	8.36	0	0.21	1.5
	Bl4QMLSSfMVEglLlL4		9.94	0	0.24	1.51
NM_000393	BiTb1Dn70e9d7THs3o	COL5A2	9.11	0.01	0.27	1.51
NM_003681	9pXn7lO6O.uJdrXEkc	PDXK	9.26	0.01	0.28	1.51
NM_005110	Z0ad.8JXpJP_tXnX5U	GFPT2	8.42	0.01	0.29	1.51
NM_145040	NA6IuyXip675eXVe58	PRKCDBP	8.65	0	0.11	1.52
NM_001078174	oHjV5_5KUuinfqfogQ	SLC29A1	9.45	0	0.2	1.52
NM_003278	3Vk2XEIHqIel0WZBWM	CLEC3B	8.12	0	0.24	1.52
NM_001122824	rTefUe71Rq5Tgu7iiU	TACC1	10.34	0.01	0.24	1.52
NM_002905	ZeVUV6x5euXlEAwkcE	RDH5	8.3	0	0.2	1.53
NM_005545	NKnCtd75._vUXyCKdY	ISLR	8.75	0	0.2	1.54
NM_001425	BgprkrXTrndXT3ee3c	EMP3	8.42	0	0.24	1.54
NM_000163	crpRMJTz0cs4cvu3pI	GHR	8.71	0.01	0.29	1.54
NM_004107	K7j1JRZeFNZT1h5wCY	FCGRT	10.4	0	0.21	1.55
NM_000803	ug564jn06h6q3XnS3o	FOLR2	8.47	0	0.24	1.55
NM_004356	cvYiWLe6kd3l9ORe18	CD81	11.04	0	0.16	1.56
NM_007021	i6SQgrik0qBqDSnqHg	C10orf10	9.04	0	0.22	1.56
NM_001393	lUj4GjV6c7v6UToFKU	ECM2	8.44	0	0.21	1.57
	E9SlZdCJ69iVeughOQ		9.29	0.01	0.29	1.57
NM_015149	Tv166Elqw5.yedk5_0	RGL1	9.76	0	0.2	1.58
NM_006169	0Qq1IgoogviEprQkrk	NNMT	8.9	0	0.21	1.58
	cRL3u0T83431EXRD18		8.64	0.01	0.27	1.58
NM_001007097	3i4kVIO74FBVRVwceE	NTRK2	9.2	0	0.17	1.59
NM_194272	rpT9IfqI4pp77T5414	RBPMS2	9.67	0	0.2	1.59
NM_001154	3uXkUVR5fX0kX8nk.s	ANXA5	11.27	0	0.21	1.59
NM_006207	xinjtCRRFUFUm3bi1g	PDGFRL	8.95	0	0.22	1.59
NM_003254	3LV5rVIyXg15Wi6CeA	TIMP1	8.72	0	0.22	1.59
NM_002076	BXu.k13vXPeV_d7tN0	GNS	10.13	0	0.23	1.59
NM_022343	TknXgre75HtFLdCc5c	GLIPR2	8.88	0.01	0.27	1.59
NM_005693	NoEh4gqQE9eonqQojU	NR1H3	8.9	0.01	0.29	1.59
	KuhFG6TjX41DSPiV6Y		8.96	0.01	0.29	1.59
NM_001753	34ROlXVXlKJ.oXDUJM	CAV1	8.82	0	0.17	1.6
NM_002178	HkHUh4rcWKp0BHcblQ	IGFBP6	8.34	0	0.21	1.6
NM_003277	BLh_l6F1a3R1JN1UpA	CLDN5	8.72	0	0.21	1.6
NM_000396	9KcIjuHEl5V4e77VKk	CTSK	10.36	0	0.24	1.6
NM_015869	0kBVz1Oe86uAd6iPdc	PPARG	8.49	0	0.19	1.61
NM_002197	fX.X0i4NNSHXToytq4	ACO1	8.74	0.01	0.26	1.61

NM_006832	o9cnRMlQCrmKiK6PFI	FERMT2	8.86	0.01	0.29	1.61
NM_000304	xyJ_nq3Ke97l1Ch7ek	PMP22	9.21	0	0.12	1.62
NM_006288	leKQlOi4hUoloR90oA	THY1	8.35	0	0.14	1.62
NM_005165	NU44rJ91ep3X35eV7c	ALDOC	9.23	0	0.22	1.62
NM_001386	ceSnuwk78Xvp_f7u3s	DPYSL2	10.63	0	0.23	1.62
NM_000093	TvUKwl2wK01RTRQJdY	COL5A1	9.33	0	0.12	1.63
NM_000138	TvxV_n_735Oj_q_iqU	FBN1	8.54	0	0.2	1.63
NM_004877	N10rh6ofoFcoXgQUIc	GMFG	9.44	0	0.24	1.63
NM_001996	0W6n1p3e6OkrTuCE0M	FBLN1	10	0.01	0.29	1.63
NM_002961	Z99V3cQV3ddJn339_s	S100A4	9.36	0	0.2	1.64
	uJNF9keCLoiLcejh6U		9.76	0	0.24	1.64
NM_018243	rJBOfrD_k3vCsoi64E	SEPT11	9.34	0.01	0.28	1.65
NM_015463	BVf4Qn1Mt9IRkT46TU	CNRIP1	9.62	0.01	0.29	1.65
NM_002508	6SXZ.isVFte0nX4XOI	NID1	8.37	0.01	0.29	1.65
NM_002448	QrVSSD4S_BInIpTt14	MSX1	8.63	0.01	0.29	1.66
NM_001645	oTiheCrhNUoql3gP1Q	APOC1	8.55	0.01	0.27	1.68
NM_001541	iiklMviV4_USFSRUkA	HSPB2	8.48	0	0.09	1.69
NM_014945	Hq4qlIqC3nu907KO0k	ABLIM3	9.49	0.01	0.27	1.69
NM_002609	Wo1Ui_tQoqIur3QxrE	PDGFRB	9.08	0	0.2	1.7
NM_015645	iteeekKDqBLp7eY0rc	C1QTNF5	9.01	0	0.23	1.7
NM_000186	folLDsE5PDJI6EoKUE	CFH	9.98	0	0.14	1.72
NM_001343	KgReX3Fd6_ojklFE1c	DAB2	8.74	0	0.15	1.72
NM_003380	QQ4tXoGUjm4DoIgf5Y	VIM	11.67	0.01	0.24	1.72
NM_001105206	uX0SnkxlH_4_OhFS4k	LAMA4	9.27	0	0.24	1.73
NM_006902	xXbV1QjvvxGKp9MGj0	PRRX1	9.68	0.01	0.29	1.73
NM_004460	ueFIFGnzWI7UYFHzEU	FAP	8.33	0	0.09	1.74
NM_001920	KtEiSRcVXXX9RF5Ad8	DCN	11.65	0	0.15	1.74
NM_199511	TpHklApjH1Ivcsp5zo	CCDC80	9.19	0.01	0.25	1.74
NM_020311	E3u67.sWkajOgYAef4	CXCR7	10.68	0	0.12	1.77
NM_000014	ZfoDn4IUQp4Auf56LU	A2M	11.13	0.01	0.29	1.78
NM_006500	onSpVBXIKlQOIg6x8o	MCAM	8.48	0	0.17	1.79
NM_004369	Zl7s26IBCIUH.09XlU	COL6A3	11.21	0	0.2	1.8
NM_001885	NtdHuDoUKASt3pXiZE	CRYAB	11.54	0	0.21	1.8
NM_001387	NoSEsN17je5ye4pJ3o	DPYSL3	11.79	0.01	0.26	1.8
NM_153370	uEk7md1eLl7snqjqj0	PI16	8.3	0	0.09	1.81
NM_173833	xKBJBHXp4QcJQzpXK0	SCARA5	8.68	0	0.1	1.83
	cqSF81At6xB7XyHjiQ		8.85	0	0.24	1.83
NM_020404	3lSUq13dBRyH6d0oHc	CD248	8.44	0	0.22	1.84
NM_002404	H6ddfnVeeOu1xVgdQA	MFAP4	9.19	0.01	0.24	1.84
NM_000552	WLnlLXeTved_5X3iVE	VWF	9.94	0.01	0.3	1.87
NM_003734	9jx.6iqBSyvihKIqCo	AOC3	9.27	0	0.2	1.9
NM_002084	6faKG5XRV0etR6fiHQ	GPX3	9.14	0	0.21	1.9
NM_019043	6Tji71IqIJNnhViZK8	APBB1IP	8.98	0	0.24	1.9

	x..cii0ZxKkrd313Xk		8.76	0	0.21	1.92
NM_002775	ZEObIyCCVRqJSjqHrY	HTRA1	10.07	0	0.1	1.95
NM_017549	flJd37V6.Xzu_Duvi8	EPDR1	8.78	0	0.19	1.97
NM_004385	NSUyuS.n3E45wKeYOo	VCAN	9.32	0	0.11	1.99
NM_003613	TVOcePpG6VUQ6RDAlU	CILP	8.55	0	0.16	1.99
	0WIk6_3_rlUHhRFVPU		9.51	0.01	0.24	1.99
	unkUFKERJfAjTpJCtI		8.22	0.01	0.24	1.99
NM_003118	QZJ1UNEckE.QufiJTk	SPARC	10.87	0.01	0.24	2.01
NM_002345	Nm.o6Q2TdIBS31Fozs	LUM	9.34	0	0.12	2.03
NM_002615	0oSSPUShGCnlXsK.Q4	SERPINF1	10.59	0	0.13	2.03
NM_001093	ByecV6BrqSIlxygO5I	ACACB	11.21	0.01	0.24	2.03
NM_000088	u1dct7Xm13tVq.SIQc	COL1A1	11.38	0	0.21	2.05
NM_002305	NISQQXuXkfQV2fQZUY	LGALS1	9.29	0	0.22	2.05
NM_145234	0i67tJX4E3XUCIcgi0	CHRDL1	9.6	0	0.24	2.05
NM_002666	uVidV71X0_AFFGuU3c	PLIN	8.18	0	0.21	2.09
NM_012193	l3uSS2p6Od7pf36tdM	FZD4	9.28	0	0.2	2.11
	upJRRddreVXTwD0b1Q		9.34	0	0.2	2.13
NM_138455	uKH_4KD656PLo75zeo	CTHRC1	10.97	0	0.14	2.17
NM_001937	9ekhFegV6onHpAd16M	DPT	11.3	0	0.2	2.17
NM_006744	NVUgmSCPsKSaSiie5c	RBP4	8.58	0	0.2	2.17
NM_144617	rX0IVB4kVnePVRJf3U	HSPB6	8.93	0	0.24	2.17
NM_000090	o0Hn7ArnXf.37T560I	COL3A1	11	0	0.17	2.3
NM_003739	ilrh6EzRdx8DW178mE	AKR1C3	9.87	0	0.14	2.33
NM_001080400	KUi5f8tn3RrpeOnQVI	KIAA1881	8.24	0	0.19	2.37
NM_016084	ZtKFRqk837e49avnuE	RASD1	10.46	0	0.21	2.38
NM_001845	TTZOhZKKkj2hRyl4Dg	COL4A1	9.9	0	0.09	2.43
NM_007069	9kJvkoOp_klOJfPotM	HRASLS3	8.72	0	0.12	2.48
NM_001928	NkEC1YkOC01HXk3r60	CFD	11.44	0	0.23	2.5
NM_012329	6p_X8jaueM_Xv1yw6k	MMD	10.18	0.01	0.27	2.5
NM_015714	xHuohSaLqKiGSyEiEg	G0S2	8.43	0	0.21	2.51
XM_001716425	uorqeiSu6V497vnfdM	FAM180B	10.89	0	0.22	2.58
NM_003251	lnelfn.xXUicIrJIt0	THRSP	8.52	0	0.2	2.6
NM_012098	EbqUp7uI76O.rHtTt4	ANGPTL2	9.51	0	0.08	2.7
NM_005276	orHi7UieXVSivCuqkA	GPD1	8.59	0	0.14	2.7
NM_003480	Z0YH4V1x1E_QT9TSEk	MFAP5	9.86	0	0.14	2.77
NM_000237	uHwCPRwJJEyR6gd6dY	LPL	8.9	0	0.21	2.86
NM_020918	NjaJ39f4yIgSVQ416c	GPAM	9.07	0	0.24	2.89
NM_000072	6uXPf6fDiHqFPrjiCk	CD36	9.44	0	0.14	3.07
NM_007085	Q.oCSr13l5wQlRuhS0	FSTL1	10.18	0	0.05	3.15
NM_013363	uSKJJzePoBeWHy5rjI	PCOLCE2	9.88	0	0.08	3.64
NM_001442	xiRTBfI6q7XrE7kgOo	FABP4	10.01	0	0.12	3.85
NM_000230	uyQvciCKeue6Uq4iec	LEP	10.7	0	0.14	5.03

Online Resource 2

List of differentially expressed genes in fascia (q<30%) fr0m microarray, fold=RH/NC.

RefSeq	nuID	Symbol	AveExpr	p	q	Fold
NM_013372	6qgEA34VJ4E7fXi0uU	GREM1	8.49	0	0	0.22
NM_000949	W1Sdf051P.BR_Xf3Uk	PRLR	7.53	0	0.27	0.39
NM_003240	HXFXlR13oh4l_VuTPw	LEFTY2	7.5	0	0.24	0.43
NM_007281	uqcH_ecovQlOXkDZd0	SCRG1	7.2	0	0.24	0.44
NM_014746	rX0uKrJxOVTl5X9.X0	RNF144A	9.68	0	0.3	0.49
	ip45H8H_6l6hTHXEik		8.18	0	0.24	0.51
NM_013377	ZijnFL9rls4_Xb0O6Y	PDZRN4	6.95	0	0.24	0.54

Online Resource 3 GO BP Conditional test for over-representation in skin microarray

GOBPID	P	OR	ExpCnt	Cnt	Size	Term
GO:0006817	0	13.053	1	9	60	phosphate transport
GO:0006820	0	6.124	2	9	117	anion transport
GO:0007584	0	10.544	1	5	39	response to nutrient
GO:0009991	0	7.589	1	6	63	response to extracellular stimulus
GO:0030199	0	30.329	0	3	10	collagen fibril organization
GO:0050878	0.001	6.449	1	6	73	regulation of body fluid levels
GO:0007275	0.001	1.993	21	35	1441	multicellular organismal development
GO:0009611	0.001	3.183	4	11	265	response to wounding
GO:0007160	0.001	6.624	1	5	59	cell-matrix adhesion
GO:0051260	0.002	14.361	0	3	18	protein homooligomerization
GO:0055088	0.002	14.141	0	3	18	lipid homeostasis
GO:0007596	0.003	7.213	1	4	44	blood coagulation
GO:0043434	0.005	10.094	0	3	24	response to peptide hormone stimulus
GO:0006629	0.005	2.285	7	15	501	lipid metabolic process
GO:0007155	0.007	2.356	6	13	437	cell adhesion
GO:0030509	0.01	7.303	0	3	32	BMP signaling pathway
GO:0050776	0.012	4.892	1	4	62	regulation of immune response
GO:0006641	0.012	14.037	0	2	12	triacylglycerol metabolic process
GO:0009612	0.012	14.037	0	2	12	response to mechanical stimulus
GO:0005996	0.012	3.871	1	5	97	monosaccharide metabolic process
GO:0031960	0.014	12.759	0	2	13	response to corticosteroid stimulus
GO:0006006	0.015	4.501	1	4	67	glucose metabolic process
GO:0007169	0.016	3.129	2	6	143	transmembrane receptor protein tyrosine kinase signaling pathway
GO:0042632	0.016	11.694	0	2	14	cholesterol homeostasis
GO:0007167	0.017	4.311	1	4	72	enzyme linked receptor protein signaling pathway
GO:0048584	0.018	4.295	1	4	70	positive regulation of response to stimulus
GO:0048519	0.019	1.798	11	19	813	negative regulation of biological process
GO:0006638	0.019	10.794	0	2	15	neutral lipid metabolic process
GO:0030168	0.019	10.794	0	2	15	platelet activation
GO:0050818	0.019	10.794	0	2	15	regulation of coagulation
GO:0018212	0.019	5.718	1	3	40	peptidyl-tyrosine modification
GO:0006869	0.019	4.167	1	4	72	lipid transport
GO:0048513	0.02	2.025	7	13	508	organ development
GO:0002253	0.02	5.567	1	3	41	activation of immune response
GO:0019217	0.021	10.022	0	2	16	regulation of fatty acid metabolic process

GO:0046486	0.021	10.022	0	2	16	glycerolipid metabolic process
GO:0006662	0.024	9.352	0	2	17	glycerol ether metabolic process
GO:0042542	0.024	9.352	0	2	17	response to hydrogen peroxide
GO:0006469	0.026	5.035	1	3	45	negative regulation of protein kinase activity
GO:0002684	0.026	3.775	1	4	79	positive regulation of immune system process
GO:0051348	0.028	4.917	1	3	46	negative regulation of transferase activity
GO:0009888	0.032	2.44	3	7	212	tissue development
GO:0043549	0.032	2.655	2	6	167	regulation of kinase activity
GO:0008283	0.032	1.877	7	13	516	cell proliferation
GO:0009719	0.034	3.45	1	4	86	response to endogenous stimulus
GO:0030334	0.038	4.312	1	3	52	regulation of cell migration
GO:0043406	0.038	4.312	1	3	52	positive regulation of MAP kinase activity
GO:0002541	0.039	7.01	0	2	22	activation of plasma proteins during acute inflammatory response
GO:0048585	0.042	6.676	0	2	23	negative regulation of response to stimulus
GO:0001501	0.045	3.141	1	4	94	skeletal development
GO:0050730	0.045	6.372	0	2	24	regulation of peptidyl-tyrosine phosphorylation
GO:0030855	0.048	6.143	0	2	25	epithelial cell differentiation
GO:0006937	0.049	6.094	0	2	25	regulation of muscle contraction

Online Resource 4 GO MF Conditional test for over-representation in skin microarray

GOMFID	P	OR	ExpCnt	Cnt	Size	Term
GO:0005201	0	21.307	1	10	48	extracellular matrix structural constituent
GO:0004857	0	5.918	2	11	161	enzyme inhibitor activity
GO:0004867	0	9.461	1	6	56	serine-type endopeptidase inhibitor activity
GO:0005518	0.001	22.997	0	3	13	collagen binding
GO:0005178	0.001	10.637	0	4	33	integrin binding
GO:0005529	0.001	5.373	1	6	93	sugar binding
GO:0030247	0.002	6.458	1	5	65	polysaccharide binding
GO:0008201	0.004	7	1	4	48	heparin binding
GO:0005515	0.004	1.711	53	67	4421	protein binding
GO:0005021	0.007	19.016	0	2	10	vascular endothelial growth factor receptor activity
GO:0005102	0.009	2.344	6	12	430	receptor binding
GO:0019199	0.01	5.04	1	4	65	transmembrane receptor protein kinase activity
GO:0005507	0.022	5.33	1	3	46	copper ion binding
GO:0005520	0.028	8.443	0	2	20	insulin-like growth factor binding
GO:0008430	0.033	7.597	0	2	22	selenium binding
GO:0005509	0.038	1.823	8	13	573	calcium ion binding
GO:0004866	0.045	6.397	0	2	27	endopeptidase inhibitor activity
GO:0004872	0.048	1.636	11	17	838	receptor activity

Online Resource 5 GO CC Conditional test for over-representation in skin microarray

GOCCID	P	OR	ExpCnt	Cnt	Size	Term
GO:0005578	0	10.222	2	13	126	proteinaceous extracellular matrix
GO:0044421	0	10.395	1	11	114	extracellular region part
GO:0005615	0	5.32	3	16	263	extracellular space
GO:0005576	0	3.663	6	18	570	extracellular region
GO:0005604	0	10.098	1	5	43	basement membrane
GO:0005581	0.002	14.644	0	3	19	collagen
GO:0031093	0.003	12.621	0	3	21	platelet alpha granule lumen
GO:0031983	0.003	11.955	0	3	22	vesicle lumen
GO:0005625	0.008	3.65	2	6	132	soluble fraction
GO:0001725	0.009	16.715	0	2	11	stress fiber

Online Resource 6 Validation of target genes *COL1A1* and *GREM1* by Quantitative RT-PCR. Quantitative RT-PCR with cDNA generated from the same total RNA samples as were used for the microarray experiments. *COL1A1* and *GREM1* were target genes. Change in gene expression is represented as fold increase in the skin and fascia from RH patients as compared to NC patients. *GAPDH* was used as an endogenous control. The y-axis depicts the expression level (-ΔCT), and the solid horizontal line denotes the group median. *COL1A1* in skin is significantly different between groups (p=0.034) as well as *GREM1* in fascia (p=0.034), using an exact Mann-Whitney test (one-sided). p values for *GREM1* were .102 in the skin and .027 in the fascia. Each sample was run in in triplicate. Note that a 1-unit change on this scale implies a 2-fold difference in expression.

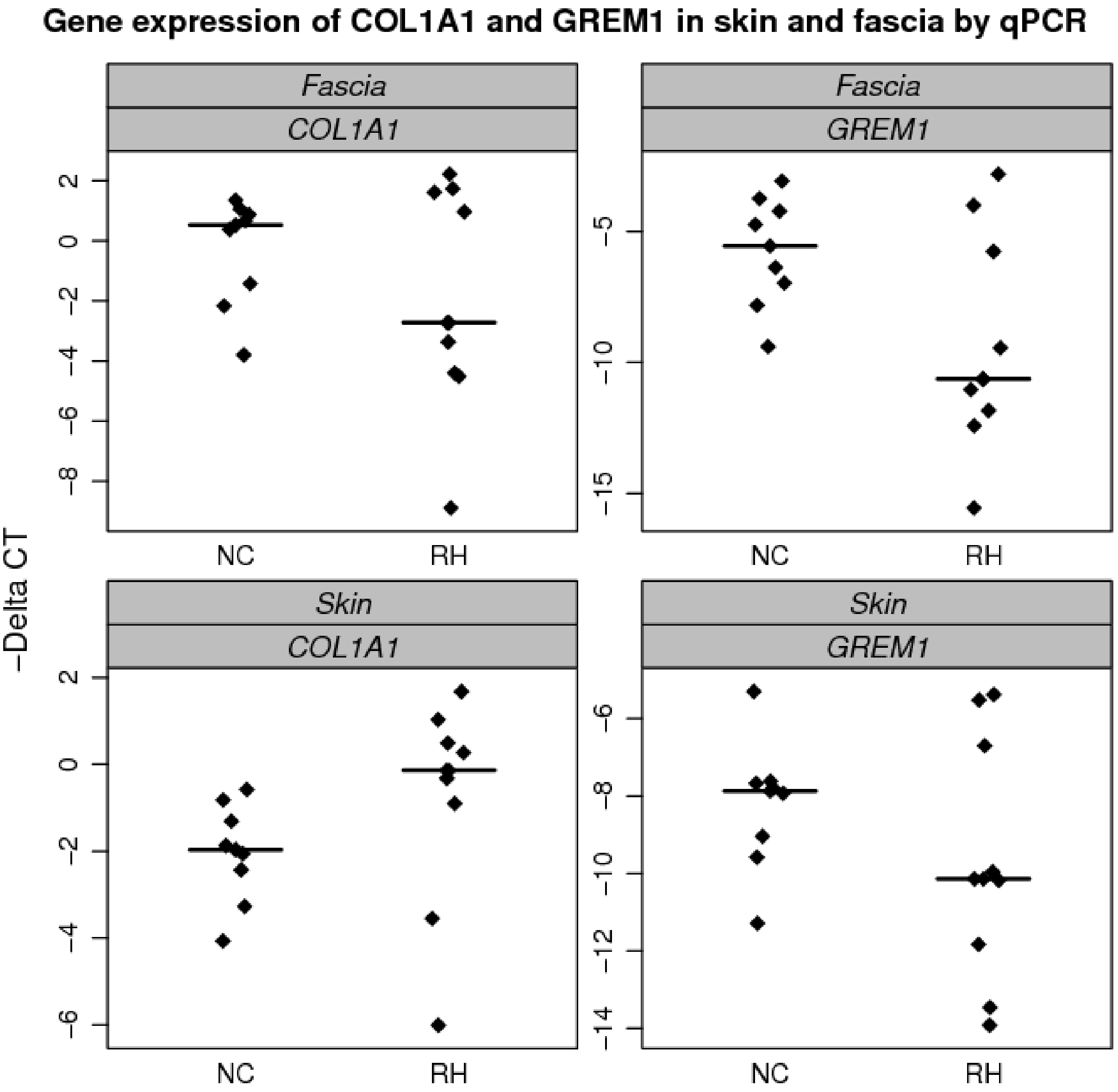

Online Resource 7 PCR array results from comparison of RH patients (n=8) and NC patients (n=7) in skin**.** Eighty expressed genes on the Human Fibrosis RT² Profiler™ PCR Array System (SABiosciences, Frederick, MD) were tested for differences via moderated t-test and raw p-values and adjusted p-values (q) are reported, along with fold change (RH/NC).

**Gene symbol**	**-ΔΔC$_t$**	**p**	**q**	**Fold change**
ACTA2	-1.317	0.088	0.290	0.401
AGT	-0.674	0.409	0.705	0.627
AKT1	-0.787	0.100	0.317	0.580
BCL2	-0.184	0.805	0.922	0.880
BMP7	0.152	0.826	0.927	1.111
CAV1	-1.544	0.019	0.159	0.343
CCL2	-0.151	0.833	0.927	0.901
CCL3	-1.356	0.070	0.275	0.391
CCR2	0.052	0.933	0.985	1.036
CEBPB	0.017	0.969	0.991	1.012
COL1A2	-1.875	0.017	0.159	0.273
COL3A1	-1.894	0.020	0.159	0.269
CTGF	-1.541	0.004	0.149	0.344
CXCR4	-0.329	0.572	0.821	0.796
DCN	-1.235	0.134	0.341	0.425
EDN1	-0.243	0.625	0.864	0.845
EGF	0.005	0.994	0.994	1.003
ENG	-1.155	0.031	0.174	0.449
FASLG	-0.305	0.727	0.884	0.809
GREM1	1.611	0.017	0.159	3.054
HGF	-1.425	0.083	0.284	0.372
IFNG	-0.418	0.395	0.705	0.748
IL10	-1.867	0.023	0.167	0.274
IL13RA2	0.343	0.610	0.860	1.268
IL1A	-1.003	0.153	0.378	0.499
IL1B	-1.511	0.109	0.332	0.351
IL4	0.143	0.763	0.899	1.104
IL5	-0.990	0.129	0.340	0.503
ILK	-0.550	0.344	0.677	0.683
ITGA1	-1.680	0.026	0.171	0.312
ITGA2	0.635	0.178	0.427	1.553
ITGA3	0.069	0.878	0.963	1.049
ITGAV	-0.325	0.656	0.867	0.798
ITGB1	-1.253	0.048	0.236	0.420
ITGB3	-1.760	0.008	0.154	0.295
ITGB5	-0.853	0.116	0.336	0.554
ITGB6	0.637	0.357	0.677	1.556

ITGB8	-0.243	0.680	0.867	0.845
JUN	-0.949	0.075	0.275	0.518
LOX	-1.853	0.007	0.154	0.277
LTBP1	-1.453	0.059	0.258	0.365
MMP1	0.455	0.432	0.71	1.371
MMP13	-0.688	0.23	0.491	0.621
MMP14	-0.242	0.673	0.867	0.845
MMP2	-1.292	0.123	0.336	0.408
MMP3	-1.315	0.064	0.267	0.402
MMP9	-1.287	0.03	0.174	0.41
MYC	0.022	0.979	0.991	1.015
NFKB1	-0.329	0.489	0.757	0.796
PDGFA	-0.447	0.247	0.513	0.734
PDGFB	-0.445	0.463	0.746	0.735
PLAT	0.188	0.804	0.922	1.14
PLAU	0.299	0.659	0.867	1.231
SERPINA1	-0.227	0.699	0.87	0.855
SERPINE1	-1.97	0.015	0.159	0.255
SERPINH1	-0.959	0.186	0.432	0.514
SMAD2	-0.302	0.537	0.813	0.811
SMAD3	0.028	0.96	0.991	1.02
SMAD4	-0.271	0.705	0.87	0.829
SMAD6	-1.225	0.122	0.336	0.428
SMAD7	-0.344	0.556	0.813	0.788
SNAI1	-0.501	0.346	0.677	0.707
SP1	0.229	0.75	0.898	1.172
STAT1	-0.055	0.911	0.985	0.963
STAT6	0.448	0.41	0.705	1.364
TGFB1	-0.389	0.426	0.71	0.764
TGFB2	-0.371	0.634	0.864	0.773
TGFB3	-0.575	0.36	0.677	0.671
TGFBR1	-1.175	0.041	0.214	0.443
TGFBR2	-1.057	0.077	0.275	0.481
TGIF1	-0.39	0.55	0.813	0.763
THBS1	-1.332	0.014	0.159	0.397
THBS2	-1.032	0.055	0.257	0.489
TIMP1	-0.594	0.38	0.699	0.662
TIMP2	-1.056	0.195	0.441	0.481
TIMP3	0.036	0.935	0.985	1.025
TIMP4	-2.812	0.002	0.149	0.142
TNF	-0.657	0.202	0.443	0.634
VEGFA	0.527	0.476	0.752	1.441

Online Resource 8 The distribution of relative gene expression in fascia and skin. The vertical lines correspond to 1.5-fold [broken] or 2-fold [solid] mean changes in relative expression. A comparison of (a) fascia and (b) skin illustrates that there is more variability in the gene fold changes for fascia than skin**.** Scales are $\log_2$.

(a)

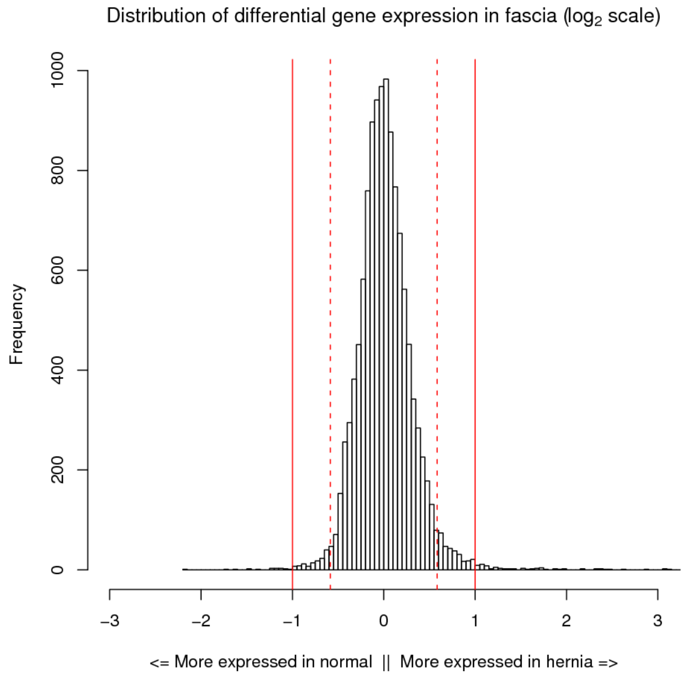

(b)

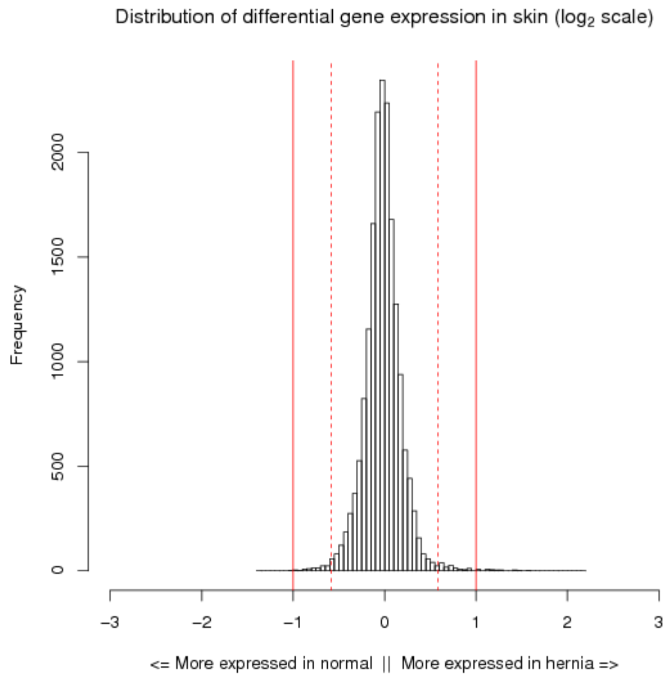

Online Resource 9 Fold change versus mean normalized signal in fascia and skin. The relationship between the fold change for (a) fascia and the background-adjusted and normalized fascia signal on a logarithmic scale is shown. A similar plot is shown for (b) skin. Hexagon binning is used to elucidate the distribution of the data. Darker shades indicate increased data density. Single blue points represent the differentially expressed genes, some of which are given in Tables 2 and 3 of the text, while the broken red lines indicate the $\log_2$ equivalent of 1.5-fold change. Scales are $\log_2$.

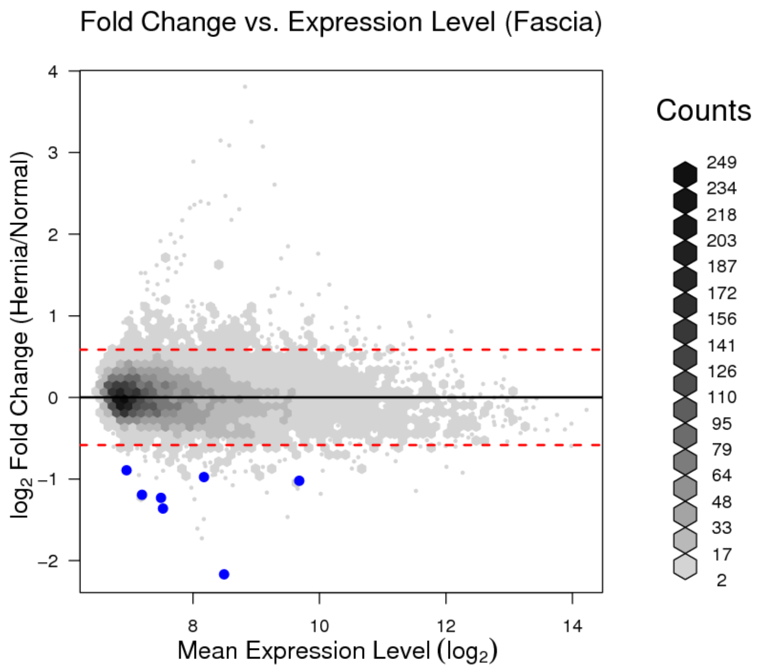

(b)

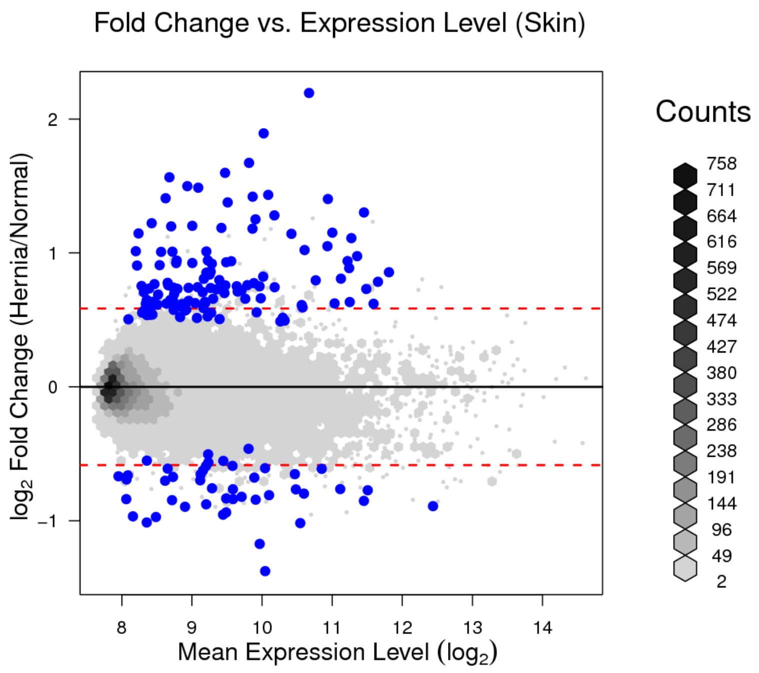

Supplement: Supplementary file 1 — Supplementary material 1 (PDF 1008 kb) [file 10029_2012_923_MOESM1_ESM.pdf]
